# A randomized, double-blind, placebo-controlled clinical trial of fluoride varnish in preventing dental caries of Sjögren’s syndrome patients

**DOI:** 10.1186/s12903-016-0296-7

**Published:** 2016-09-23

**Authors:** Weini Xin, Katherine Chiu Man Leung, Edward Chin Man Lo, Mo Yin Mok, Moon Ho Leung

**Affiliations:** 1 Dental Care Center, The No.1 Affiliated Hospital of Shantou Medical College, Shantou, China; 2 Prosthodontics, Faculty of Dentistry, The University of Hong Kong, Hong Kong SAR, China; 3 Dental Public Health, Faculty of Dentistry, The University of Hong Kong, Hong Kong SAR, China; 4 Department of Biomedical Sciences, City University of Hong Kong, Hong Kong SAR, China; 5 Department of Medicine, Queen Elizabeth Hospital, Hong Kong SAR, China

**Keywords:** Sjögren’s syndrome, Fluoride varnish, Dental caries, Oral Candida, Oral lactobacilli

## Abstract

**Background:**

Sjögren’s syndrome (SS) patients are prone to caries development due to reduction of salivary flow. Topical fluoride is commonly prescribed for caries prevention.

**Methods:**

In this 24-month randomized, double-blind, placebo-controlled clinical trial, SS patients were randomly assigned to receive either fluoride varnish or placebo gel quarterly. Development and arrest of caries at the coronal and root surfaces were recorded at 12-month and 24-month and compared to that of the baseline. Effect of fluoride varnish on oral Candida and lactobacilli colonization was explored by comparing baseline oral microbiological assessments to data obtained at 12-month and 24-month.

**Results:**

Seventy-eight SS patients (mean age = 50 years, 2 men) completed this trial. At 24-month, the mean new coronal enamel caries were 1.6 surfaces in both groups, and new dentin caries were 1.4 and 2.7 surfaces in the fluoride and placebo group respectively (*p* > 0.05). Mean arrested caries were 0.6 and 0.7 surfaces for fluoride and placebo groups respectively and that of root caries were 0.3 and 0.1 surfaces (*p* > 0.05). The mean oral Candida count was reduced by 30 % in the fluoride group but increased 61 % in the placebo group while no change in oral lactobacilli counts in both groups at 24 months (*p* > 0.05). SS patients receiving fluoride varnish were significantly less likely to develop dentin caries (*p* < 0.05). In contrast, those with high baseline DMFS scores (*p* = 0.05), harbored mixed Candida species (*p* < 0.05), or unstimulated whole saliva at low pH (*p* < 0.01) were significantly more likely to develop dentin caries.

**Conclusions:**

Results of this randomized clinical trial did not provide clear evidence to support or refute that quarterly applications of fluoride varnish can prevent development of dental caries in people with Sjögren’s syndrome.

**Trial registration:**

This study was retrospectively registered at the ISRCTN registry (ISRCTN85164658) on 9 Sept 2016 and was funded by the Research Grant Council of Hong Kong.

## Background

Sjögren’s syndrome (SS) is a slowly progressive autoimmune disorder of exocrine glands which is characterized by intense lymphocytic infiltration [1]. Salivary and lacrimal glands are the most commonly affected exocrine glands, resulting in reduced secretion of saliva (hyposalivation) and tears [2].

SS patients often complain of dry mouth (xerostomia). Reduction in salivary flow rate and high caries occurrence have also been reported in previous studies [3]. In addition, there is a high prevalence of SS sufferers presented with oral colonization of pathological micro-organisms such as lactobacilli and Candida [4].

Prevention of dental caries in SS patients is important. Topical fluoride has been extensively used to prevent caries in children and elderly people, however, little evidence is available for its efficacy in SS patients at present. Most fluoride varnishes contain 22600 ppm fluoride. It has been reported to effectively prevent enamel caries and dentin caries, and to arrest initial caries lesions in children and adolescent [5, 6]. The Sjögren’s Syndrome Foundation Clinical Practice Guidelines Committee strongly recommends the use of topical fluoride [7]. The American Dental Association (ADA) suggests that people at moderate risk should receive fluoride varnish or gel applications at 6-month intervals and higher-risk patients at three- to 6-month intervals [8].

The current study reports a randomized, double-blind, placebo-controlled clinical trial with the primary aim of investigating the efficacy of fluoride varnish in preventing dental caries in Chinese patients with SS. The effect of fluoride varnish on oral colonization of Candida and lactobacilli in Chinese patients with SS over a 24-month period will also be investigated.

## Methods

### Study design and participants

This was a parallel study. Randomized, double-blind, placebo-controlled clinical trial design which lasted for 24 months was employed. Dental records of 15 SS patients were randomly selected from those who had participated in previous consecutive studies revealed a mean caries increment of 3.5 surfaces (SD = 2.8) over 24 months [3]. Assuming the caries preventive fraction of fluoride varnish was 35 % [9], the estimated 24-month mean caries increment of the fluoride group would be 2.3 surfaces (SD = 1.8). The estimated minimum sample size was 48 per group to achieve α = 0.05 and β = 80 %. Allowing for a 10 % annual dropout, 120 SS patients was planned.

SS patients were recruited from the Rheumatology Clinics of the Queen Mary Hospital (QMH) and Queen Elizabeth Hospital (QEH), Hong Kong. These two hospitals were selected because they were the two largest public hospitals in Hong Kong and are within 30 minutes travelling time to the Prince Philip Dental Hospital (PPDH). Patients from QMH and QEH are comparable in terms of ethnic origin, diet and culture. Diagnosis of SS was established using the American-European Consensus Group (AECG) criteria [10] at least 6 months before the commencement of this study. Potential recruits were excluded if they were under 18 years of age, had less than 8 natural teeth, had severe periodontal disease (e.g., periodontal pockets were deeper than 6 mm at two or more sextants), had received therapeutic irradiation to the head and neck region, had concurrent systemic illness (except connective tissue disorder associated with sSS), were taking medication that altered salivary flow, or had participated in a clinical trial within 6 months before commencement of this trial. Socio-demographic information regarding education level, employment status, and oral hygiene measures were obtained. To ensure comparability of the characteristics of the subjects in the intervention and control groups, a stratified block randomization method with a block size of four was applied so that a similar number of pSS and sSS subjects were allocated to each group [11]. Concealment of participant allocation was kept by a clinical staff who was not involved in the trial. Topical applications of fluoride varnish or placebo gel were carried out by one hospital dentist who was not involved in research team. Blinding of the participants and the examiner was maintained until all clinical data were collected at 24-month.

All recruited subjects were invited to attend a pre-baseline full-mouth oral and dental examination at the PPDH. Bitewing radiographs were taken to aid diagnosis of caries. Standard oral hygiene instruction on toothbrushing and flossing was given and participants were advised to brush twice a day and floss every day. Dental treatments including scaling, tooth extraction, root canal treatment, periodontal therapy and dental prosthesis were provided if indicated. Restorations were provided except for initial caries lesion and arrested non-cavitated caries lesions. The use of fluoride-releasing restorative materials was avoided. All treatments were completed at least 14 days before commencement of the baseline data collection.

### Clinical procedure

All clinical procedures were carried out between 9:00 am and 12:00 noon in a surgery room at the PPDH. Participants were advised to refrain from food or beverage intake (except water) one hour before the attending dental examination. They were asked to rinse their mouth with 10 ml of sterile phosphate-buffered saline (PBS; 0.01 M; pH = 7.2) for 30s prior to collection in a sterile container. Dentures, if present, were retained during rinsing. The oral rinse sample was then sent for microbiological analysis. After resting for 5 min and voiding the mouth, a sterile, pre-weighed plastic vial was provided for collection of unstimulated whole saliva (UWS). Over a period of 5 min, all saliva was drained into the vial by drooling or gentle spitting. For stimulated whole saliva (SWS) collection, subjects were instructed to chew on a piece of sterilized silicone rubber tubing (2 mm × 8 mm∅) and to spit all the saliva into another sterile, pre-weighed plastic vial for 5 min. The weights of vials were recorded and UWS and SWS flow rates were determined and expressed as g/min. Immediately after saliva collection, pH values and buffer capacity of UWS and SWS were determined at chair side using a pH meter (Sentron 501 Pocket FET pH meter, Sentron, WA, USA) and a commercial strip (CRT® buffer, Vivadent, Liechtenstein) respectively. Supra-gingival dental plaque was collected from all accessible coronal tooth surfaces of the whole mouth using a sterile sickle scaler and then dispersed into a pre-weighed vial containing 500 μl PBS.

The examiner was trained and calibrated before the start of the baseline examination and recalibrated at 6-month, 12-month, 18-month and 24-month. The examiner received training through the “ICDAS E-Learning Course” in the ICDAS official website. After calibration, data collection was performed by the calibrated examiner. To assess intra-examiner reliability, duplicate examinations were carried out on one in ten subjects.

The Silness-Löe plaque index [12] was modified where score 0 denoted no plaque accumulation, and score 1 dental plaque accumulation when a WHO probe (Guilin Woodpecker Medical Instrument Co., Ltd) was run along the gingival margins of the index teeth. Plaque index was expressed as the percentage of examined sites with plaque accumulation.

After air-drying and plaque removal, all exposed coronal and root surfaces were evaluated using a WHO probe according to the modified International Caries Detection and Assessment System (ICDAS) [13]. Molars and premolars were considered to have five coronal surfaces and canines and incisors were considered to have four. Codes were given as follows: sound (code 0), visual change on enamel (code 1), distinct visual change on enamel (code 2), caries extended to dentin (code 3), filled without decay (code 4), filled with decay (code 5), arrested caries (code 6), missing (code 7), crowned (code 8). Decayed, missing, or filled surface (DMFS) score at baseline was computed by summing the number of caries, missing and filled surfaces related to caries. The exposed root surfaces were coded as sound (code 0), no cavitation with discolored area (code 1), cavitation with discolored area (code 2), or arrested caries (code 6). Dental examination was carried out at 12-month and 24-month.

### Microbiological assessment

Lactobacilli were explored in the SWS and dental plaque samples. The SWS and dental plaque samples were vortexed for 30s at maximum setting (Autovortex Mixer SA2, Stuart Scientific, London, UK) and then serially diluted (10^−2^ and 10^−3^) with 0.01 M sterile PBS (pH = 7.2). A 50-μl aliquot of the 1:100 and the 1:1000 dilutions were duplicately spiral-plated (Spiral Systems Autoplate 4000 Spiral plater, Spiral Biotech, Norwood, Mass) on Rogosa agar (Difco, Detroit, MI, USA). The Rogosa agar plates were incubated for 3 days in an anaerobic chamber (37 °C, 5 % CO_2_ and 95 % N_2_). Following incubation, a stereomicroscope (Olympus TL, Tokyo, Japan) was used to verify the presence of colonies. The colonies were subcultured using blood agar plates (Oxoid, Basingstoke, Hampshire, UK), which contained 5 % defibrinated blood, 0.0005 % (5 mg/L) haemin and menadione, to obtain pure isolates for identification. Identification for lactobacilli was confirmed by colony morphology, gram-stain reaction, Candida was explored in the oral rinse and dental plaque samples.

Candida were explored in the oral rinse and dental plaque samples. The samples were vortexed for 30s at maximum setting and 1:10 diluted with 0.01 M sterile PBS (pH = 7.2). To estimate the level of Candida in each sample, a 50-μl aliquot of the undiluted (oral rinse samples) and dilution samples (oral rinse and dental plaque samples) were duplicately spiral-plated by using the spiral plater onto the Sabouraud dextrose agar (SDA; Oxoid, Unipath Ltd, Basingstoke, Hampshire, UK) and CHROMagar (Becton Dickinson Europe, Le Pont de Claix, France). All plates were incubated for 2 days at 37 °C in air. After incubation, plates with appropriate number of colonies which were well separated and evenly dispersed were chosen for counting. The number of the colony in oral rinse and dental plaque samples were recorded and expressed as CFU/ml. The selected Candida colonies were subcultured on SDA plates to obtain pure isolates. Candida was speciated and identified based on the followings: colony morphology in CHROMagar and SDA, cell morphology, gram-stain reaction, and result of the API ID 32C system (bioMérieux, France) and RapID-ANA II system (Remel, Lenexa, KS, USA).

Counts of lactobacilli and Candida were expressed as colony forming units per milliliter (CFU/ml) in salivary samples and colony forming units per gram in dental plaque samples.

### Study interventions

In the test group, topical fluoride varnish (5 % NaF varnish, Duraphat®, Colgate, Colgate-Palmolive Company, New York, USA) was applied; while in the control group, a water-based gel without fluoride was used as placebo (K-Y® Gel, McNEIL-PPC Inc., Fort Washington, USA). A trained clinician applied the fluoride varnish/placebo gel that was dispensed by a dental surgery assistant in single-dose vials with the name and record number of the subjects pre-labelled [14]. All teeth were dried and isolated, and fluoride varnish/placebo gel was applied using a micro-brush to all teeth surfaces. Participants were asked to avoid drinking and eating during the first two hours after application and to avoid eating abrasive food on that day. The dental surgery assistant escorted the patients to leave the clinics after application of the agent, so as to avoid the contact between patients and the examiner or the other patients in this study.

Follow-up visits were arranged every 3 months after baseline for applications of fluoride varnish/placebo gel up to 24 months. Dental condition and oral hygiene condition were recorded at the 12-month and 24-month visits, as well as oral rinse, salivary and dental plaque samples collection, and measurement of the relevant index (pH, buffering capacity and microbiological profiles).

### Statistical analysis

Differences in continuous variables between the fluoride and placebo groups were tested by *t*-test or Mann-Whitney *U* test for data which were normally or not normally distributed respectively. Differences in categorical variables between groups were tested by Chi-squared tests or Fisher’s exact tests whichever was appropriate. Repeated measures analysis of variance or Friedman test, whichever was appropriate, was performed for evaluating differences among the same continuous variables of three-time point assessments (baseline, 12-month and 24-month). Pearson’s correlation or Spearman’s rank correlation, whichever was appropriate, was used to explore associations between parameters of interest.

Multivariate analysis of estimation of parameters in occurrence of new caries increments is appropriate for this dataset. The logistic model was applied to predict the occurrence of caries over the 24-month period and to explore the effectiveness of intervention. Potential risk factors associated with development of new caries lesions was assessed by odds ratio through logistic regression analyses. Variables with a *p*-value of 0.10 or below were entered into regression model (Forward selection) for determination of significant factors. The level of significance was set at 5 % (*p* < 0.05). Data analysis was processed using SPSS software (version 20.0).

## Results

### Recruitment and socio-demographic characteristics of participants

**Fig. 1:**
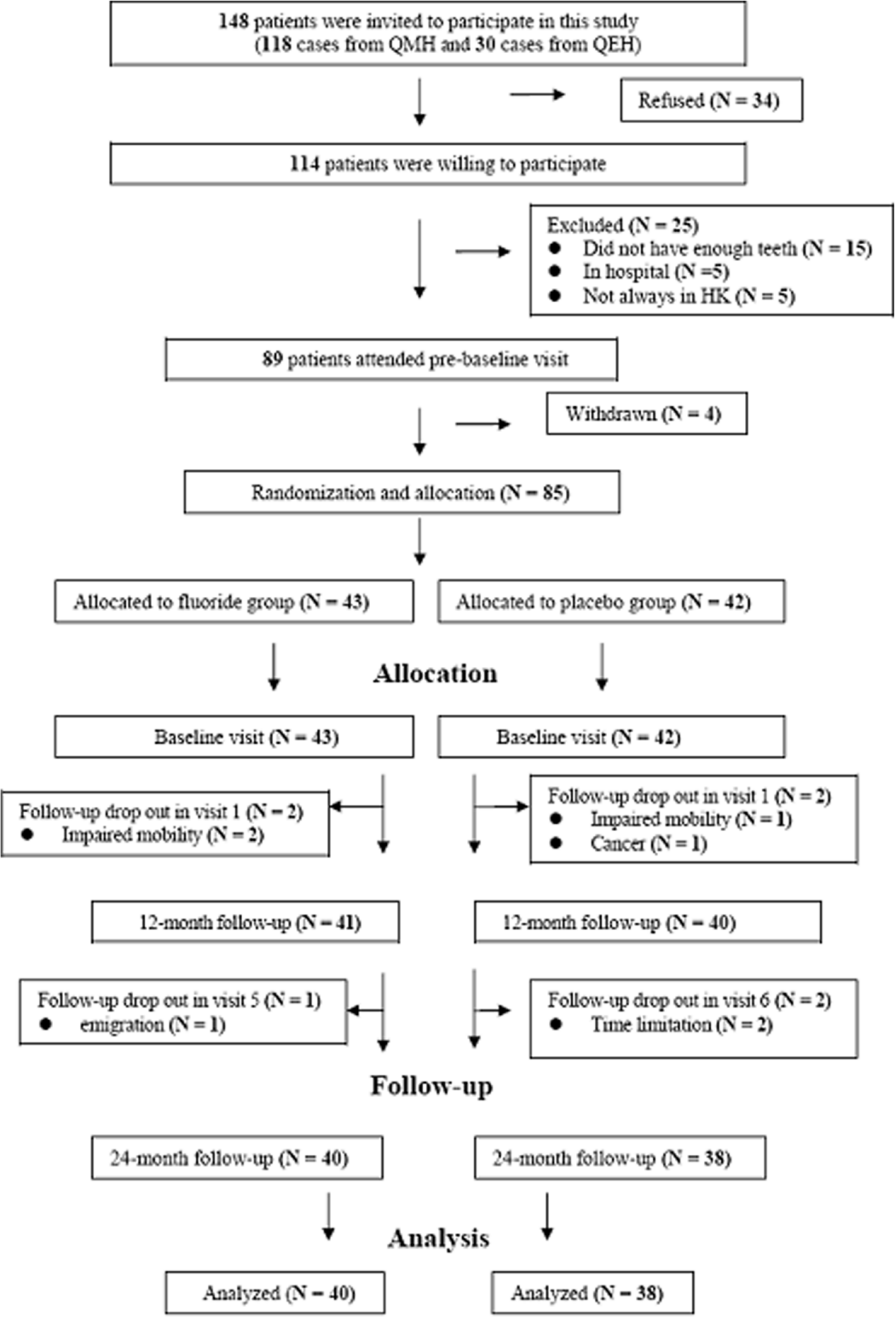
CONSORT flow diagram of this randomized trial

The flow of the study is presented in Fig. 1. Among 114 recruited SS patients, 85 patients met the inclusion criteria and received prebaseline treatment. They were randomly allocated into the fluoride group (*N* = 43) and the placebo group (*N* = 42). During the follow-up period, seven patients (3 in the fluoride group and 4 in the placebo group) withdrew. No significant difference was found between the seven drop-outs and the remaining 78 patients (35 pSS and 43 sSS), who were included for data analysis. Out of the 78 patients, 76 (97 %) were women and the mean age was 50 years (SD = 9.9). The average duration of diagnosis was 101.3 months (SD = 78.6) (Table 1). More than 85 % of the subjects brushed their teeth twice or more per day and used fluoride toothpaste. Approximately 75 % of them practised interdental cleaning using dental floss or interdental brush, while only one-third of them used fluoride mouthrinse. There was no significant difference between subjects in fluoride and placebo groups in terms of their regular oral hygiene habits and use of fluoride products (*p* > 0.05), except the frequency of tooth brushing and the use of fluoride toothpaste (*p* < 0.05).There was no statistical significant difference in the type of SS, gender, age, duration since diagnosis, education level or work status between the two groups (*p* > 0.05).

**Table 1:** Socio-demographic features, oral hygiene practice and baseline condition by group

	Fluoride group (*N* = 40)	Placebo group (*N* = 38)	*p*-value
Type of SS (primary: secondary) (%)	45.0 : 55.0	44.7 : 55.3	N.S.
Gender (Female: Male) (%)	97.3 : 2.7	97.4 : 2.6	N.S.
Age at baseline (years)			
Mean (SD)	49.2 (9.1)	50.9 (10.8)	N.S.
Range	29–71	26–75	
Time since diagnosis (months)			
Mean (SD)	117.5 (97.1)	84.2 (48.4)	N.S.
Range	3–425	2–177	
Education level (%)			
Primary school or below	30.0	21.1	N.S.
Secondary school or above	70.0	78.9	
Employed (%)	62.5	44.7	
Oral hygiene practice (%)			
Brushed twice or more per day	100.0	89.5	**0.001**
Practised interdental cleaning	70.0	78.9	N.S.
Daily use of fluoride (%)			
Toothpaste	87.5	100.0	**<0.001**
Mouthrinse	37.5	34.2	N.S.
Three meals or less per day (%)	90.0	89.5	N.S.
*Saliva*			
Unstimulated whole saliva			
Mean flow rate (SD), range	0.22 (0.24), 0–0.99	0.26 (0.26), 0–1.12	N.S.
Mean pH (SD), range	7.2 (0.4), 6.3–8.3	7.3 (0.5), 6.3–8.5	N.S.
Stimulated whole saliva			N.S.
Mean flow rate (SD), range	0.48 (0.58), 0–2.60	0.51 (0.47), 0–1.73	N.S.
Mean pH (SD), range	7.8 (0.4), 6.8–8.6	7.7 (0.5), 6.5–8.5	N.S.
*Dental condition*			
Mean number of teeth (SD), range	26.6 (3.0), 18–32	26.7 (3.8), 12–32	N.S.
Mean DMFS (SD), range	45.6 (18.8), 4–78	41.2 (23.1), 1–114	N.S.
Mean DS (SD), range	0.9 (1.1), 0–4	0.9 (0.9), 0–4	N.S.
Mean MS (SD), range	25.0 (13.7), 0–65	25.7 (18.6), 0–94	N.S.
Mean FS (SD), range	19.8 (12.5), 0–54	14.7 (10.6), 0–43	0.06
Mean plaque index (SD) (%)	28 (25), 0–88	33 (26), 4–100	N.S.
Removable partial denture wearers (%)	7.5	13.2	N.S.

*N.S.* not significant

### Salivary flow rates, saliva pH, saliva buffering capacity and dental conditions

Mean plaque index scores, DMFS, salivary flow rate and pH of both groups at baseline were shown in Table 1. There was no significant difference in these parameters between the two groups (*p* > 0.05).

The mean salivary pH values over 24 months in fluoride group and placebo group were within the normal range. No statistically significant difference existed between the salivary flow rates, pH value and buffer capacity of the fluoride group and placebo group (*p* > 0.05).

Most subjects developed caries with time in both groups (Table 2). At 12-month, enamel caries accounted for 63.6 % of the caries lesions while at 24-month, enamel and dentin caries lesions were equally detected. There was no significant difference in the distribution in enamel lesions, dentin caries lesions and arrested caries lesions between the two groups at 12-month and 24-month visits (*p* > 0.05). The mean number of caries lesions developed in the maxillary and mandibular jaws showed no statistically significant difference (*p* > 0.05). More anterior teeth in the placebo group developed dentin caries lesions than those in the fluoride group (*p* < 0.05), but such difference was not observed in premolars and molars (*p* > 0.05). More distal surfaces in the placebo group developed dentin caries lesions than those in the fluoride group (*p* < 0.05), however the difference was not observed in the other surfaces. The difference in the root caries increments between two groups was not statistically significant (*p* > 0.05). Comparing the difference of the numbers of dentin caries lesions at 24-month, the caries preventive fraction of fluoride varnish in SS patients is 33.3 %. Intra-examiner reliability was 0.96 and 0.90 for coronal caries and plaque index respectively.

**Table 2:** Different types of lesions at the subject and surface levels

	12-month			24-month		
Group	Fluoride group (*N* = 40)	Placebo group (*N* = 38)	*p*-value	Fluoride group (*N* = 40)	Placebo group (*N* = 38)	*p*-value
Number of subjects (%)						
No caries	15 (37.5)	19 (50)	N.S.	11 (27.5)	12 (31.8)	N.S.
Enamel lesion	19 (47.5)	15 (39.4)	N.S.	21 (52.5)	18 (47.4)	N.S.
Dentin lesion	4 (10)	8 (21.1)		13 (32.5)	16 (42.1)	
Arrested lesion	7 (17.5)	5 (13.2)		10 (25.0)	14 (37.5)	
Number of surface						
Enamel lesion						
Sum	41	34	N.S.	64	60	N.S.
Mean (SD)	1.0 (1.5)	0.9 (1.5)		1.6 (2.3)	1.6 (2.5)	
Range	0–6	0–7		0–10	0–11	
Dentin lesion						
Sum	8	35	N.S.	55	78	N.S.
Mean (SD)	0.2 (0.6)	0.9 (2.4)		1.4 (2.6)	2.1 (4.2)	
Range	0–3	0–11		0–11	0–22	
Arrested lesion						
Sum	17	13	N.S.	22	25	N.S.
Mean (SD)	0.4 (1.2)	0.3 (1.2)		0.6 (1.3)	0.7 (1.3)	
Range	0–6	0–6		0–7	0–7	

*N.S.* not significant

DMFS at baseline correlated positively to the number of new dentin caries developed at 24-month in the fluoride (r_s_ = 0.43, *p* = 0.005) and the placebo (r_s_ = 0.37, *p* = 0.02) groups. In addition, F-component of DMFS at baseline showed a positive correlation to the number of new dentin caries at 24-month in the fluoride (r_s_ = 0.40, *p* = 0.01) and the placebo (r_s_ = 0.36, *p* = 0.03) groups.

As less than the original estimated sample size was recruited, post-hoc power calculation was conducted. According to the initial sample size determination, 38 subjects per group would provide 80 % power to detect a preventive fraction of 42.8 %, i.e., estimated 24-month mean caries increment of fluoride group would be 2 surfaces (SD = 1.6).

Intra-examiner reliability was 0.96 for coronal caries and 0.90 for plaque index.

### Microbiology

The mean counts of lactobacilli and Candida at the various time-points and sites were shown in Table 3. There was no statistically significant difference in the prevalence of species and the mean counts of lactobacilli and Candida between the two groups at baseline and 24-month (*p* > 0.05). Correlations (r_s_) between the mean counts of the microbes in the saliva and dental plaque and the mean salivary flow rates and new various lesions were presented in Table 3.

**Table 3:** Correlations (r_s_) between the mean numbers of species carried, mean counts of *Candida* in the oral rinse samples and dental plaque samples and the mean salivary flow rates and new caries lesions

		Fluoride group (*N* = 40)		Placebo group (*N* = 38)	
Saliva samples					
		Lactobacilli	Candida	Lactobacilli	Candida
Mean counts (×10^4^ CFU/ml) (SD)	Baseline	1.6 (4.6)	591.5 (1705.1)	8 (25.8)	184.3 (369.3)
	Over 24-months	6.3 (14.2)	428.3 (828.2)	13.7 (45.6)	291.6 (541.1)
Mean counts					
*Correlations (r*_*s*_ *)*		Lactobacilli	Candida	Lactobacilli	Candida
Mean salivary flow rates	Unstimulated whole saliva	−0.37*	−0.67**	0.44**	−0.77**
	Stimulated whole saliva	−0.38*	−0.69**	−0.39*	−0.77**
New lesions (over 24 months)	Enamel lesion	−0.03	−0.19	0.17	−0.02
	Dentin lesion	0.40**	0.53**	0.35*	0.56**
	Arrested lesion	0.25	0.44**	0.24	0.32
Dental plaque samples					
		Lactobacilli	Candida	Lactobacilli	Candida
Mean counts (×10^6^ CFU/g) (SD)	Baseline	15.8 (62.5)	60.2 (172.9)	1.7 (6.3)	9.5 (20.4)
	Over 24-months	56.6 (178.9)	37.2 (73.5)	119.1 (444.6)	40.9 (164.0)
Mean counts					
*Correlations (r*_*s*_ *)*		Lactobacilli	Candida	Lactobacilli	Candida
Mean salivary flow rates	Unstimulated whole saliva	0.51**	−0.53**	−0.69**	−0.75**
	Stimulated whole saliva	−0.48**	−0.58**	−0.69**	−0.79**
New lesions (over 24 months)	Enamel lesion	0.11	−0.05	0.04	−0.04
	Dentin lesion	0.44**	0.45**	0.47**	0.48**
	Arrested lesion	0.26	0.38*	0.33*	0.32

* Significant Spearman’s correlation *p* < 0.05

** Significant Spearman’s correlation *p* < 0.01

The most predominantly isolated lactobacilli were *L. fermentum*. Others lactobacilli species isolated included *L. acidophilus*, *L. minutis* and *L. casei.*

*C. albicans* was the most frequently isolated Candida species, other Candida species such as *C. dubliniensis*, *C. tropicalis*, *C. glabrata*, *C. sake* and *C. parapsilosis*. More than 10 % of patients in both groups had mixed Candida colonization in saliva and dental plaque samples, usually *C. albicans* with another Candida species.

The odds ratio of SS patients receiving fluoride varnish who developed dentin caries was 0.22 (Table 4). The odds ratios per unit increase in the mean number of Candida species in dental plaque and mean UWS pH of dentin caries development were 5.189 (95 % CI: 1.307, 20.602) and 0.052 (95 % CI: 0.006, 0.414) respectively. Logistic regression analysis revealed that SS patients who received placebo (*p* < 0.05), presented with low UWS pH (*p* < 0.01) and had high baseline DMFS level (*p* = 0.050) were significantly more likely to develop dentin caries when compared to their counterparts.

**Table 4:** Final logistic regression model for the occurrence of new dentin caries lesions in SS patients over a 24-month period

		Adjusted		
		Odds ratio	95 % confidence interval	
Factors	*P*-value		Lower	Upper
Intervention (Ref. = placebo group)	0.035	0.221	0.054	0.901
Mean number of *Candida* species in the dental plaque samples	0.019	5.189	1.307	20.602
Mean pH of unstimulated whole saliva over 24 months	0.005	0.052	0.006	0.414
DMFS (at baseline)	0.05	1.033	1.000	1.067

SS patients who had high UWS buffering capacity (*p* < 0.05) and harbored high level of Candida in the oral rinse samples (*p* < 0.05) were significantly more likely to have caries arrested at 24-month as compared to their counterparts (Table 5). Those with high UWS buffer capacity were five times as likely as those with low or medium UWS buffer capacity to have arrested caries lesions.

**Table 5:** Final logistic regression model for the occurrence of new arrested caries lesions in SS patients over a 24-month period

		Adjusted		
		Odds ratio	95 % confidence interval	
Factors	*P*-value		Lower	Upper
Intervention (Ref. = placebo group)	0.054	0.315	0.097	1.022
Buffering capacity of unstimulated whole saliva at baseline (Ref. = medium and low)	0.017	5.018	1.339	18.804
Mean counts of *Candida* in oral rinse samples over 24 months	0.015	1.002	1.000	1.003

## Discussion

SS patients who received quarterly applied fluoride varnish developed 32.5 % less coronal dentin caries than those who received a placebo, which was similar to the result obtained in a study on hyposalivated patients who self-administered 0.05 % NaF mouthrinse 3 times daily [15]. The caries preventive fraction of fluoride varnish in SS patients is similar to that of other groups of subjects [16]. Although subjects in the placebo group developed higher mean new caries lesions than the fluoride group, the differences were not statistically significant. Type II error may be responsible for the apparent lack of difference in caries increments between the two groups as less SS subjects were recruited than estimated. On the contrary, when other confounding factors were controlled, the odds ratio of dentin caries development was significantly lower in the fluoride varnish group than the placebo group. Such contradicting findings imply a lack of clear evidence to support or refute the hypothesis that quarterly applied fluoride varnish can prevent caries development in this group of patients. Nevertheless, it is interesting to note that SS subjects in this study were generally having high dental awareness and their level of oral hygiene was satisfactory. Caries experience as reflected in the number of standing teeth and DMFS is even comparable to some non-SS subjects in other studies [17, 18]. In addition, more than 30 and 50 % of them presented with normal range of UWS and SWS flow rates respectively. Salivary flow rate, UWS in particular, has been shown to negatively associate with caries development [19].

In this study cohort, both the number of root caries and root caries increment were low. An earlier cross-sectional study of SS patients has revealed a mean decayed or filled root surface of 5.3 which was 10 times as that in the current study cohort [20]. Caries increments in both groups were much lower than the estimated mean annual root caries increment in the general older population [21]. Hence, efficacy of fluoride varnish in root caries prevention in SS patients cannot be discerned.

Reduced salivary flow rate leads to changes in some specific components of saliva, including the immunoglobulins (secretory IgA, IgG, and IgM), antibacterial protein (lysozyme, lactoferrin, peroxidases) and the concentrations of electrolytes (sodium, chloride, and phosphate) [17]. In addition, reduced salivary output may hinder mechanical flushing of microorganisms from teeth surfaces [22]. Therefore, antibacterial activity of saliva is decreased and bacteria can flourish in the mouth of SS patients. Despite the fact that salivary pH of most SS subjects in this study was within normal range, a negative correlation between salivary pH and development of new dentin lesions was detected and low UWS pH was found to be a strong predictor of dentin lesions development. Rates of the demineralization and remineralization of dental hard tissues are pH-mediated [23]. At neutral pH (7.0), saliva is supersaturated with calcium phosphate. However, when the pH decreases to a point where the saliva ceases to be supersaturated, the hydroxyapatite crystals begin to dissolve and demineralization occurs. It has been reported that UWS pH in SS patients can be as low as only one tenth of a pH unit above the mean critical pH [17]. A minute drop in pH, hence, can lead to undersaturation of hydroxyapatite that results in either caries lesions or erosive damage to the teeth depending on the origin of the acidic challenge. In addition, acidic oral environment is highly suitable for the survival and proliferation of cariogenic bacteria. Acidic saliva has poor ability to maintain oral homeostasis. When the saliva pH level decreases, the risk of dentin caries development increases.

Buffering capacity of saliva is in fact more critical than its pH value in oral homeostasis [24]. Results of the logistic regression revealed that occurrence of new arrested caries lesions was related to the level of the buffering capacity of UWS. Arrested lesions are the result of remineralization of a demineralized lesion and the buffering capacity of saliva plays a critical role in helping to restore a neutral pH at the tooth surface and to increase the rate of remineralization [25]. Although some studies showed that the salivary bicarbonate and phosphate concentrations of SS patients were lower than those of healthy subjects [17], in this study, more than 80 % of SS patients had medium to high salivary buffering capacity, hence enamel and dentin caries lesions in these patients can be remineralized and became arrested lesions.

The oral environment of SS patients does not favor the growth of S. mutans because the saliva pH was higher than the optimal pH that supports its growth. Furthermore, S. mutans and Candida species do not coaggregate well [26]. The high oral prevalence of Candida (55–65 %) may prevent the colonization of S. mutans in this study population. The result suggests that fluoride varnish has little effect on the level of lactobacilli in SS patients, which is in agreement with that reported in other studies [27]. Some study found that NaF at one mmol/L could only inhibit the growth of less than half of oral lactobacilli species [28]. The low concentration of fluoride (1.2 mmol/L NaF) in fluoride varnish cannot inhibit the growth of oral lactobacilli. Furthermore, the concentration of fluoride cannot be maintained as it would gradually decline with time after application. An in-vitro study has demonstrated that fluoride varnish could release fluoride for five to 6 months and the mean concentration was about 0.025 mmol/L [29].

In this study, Candida counts were found to be positively correlated to the number of new dentin caries lesions. Candida produces proteolytic enzymes which can break down the organic collagen matrix of dentin [30]. Furthermore, the penetrating power of hyphae from enamel into dentin seems to support that Candida has a crucial role in dental caries development [31]. Mixed colonization of Candida spp. was observed in about 10 % of the SS subjects which generally occurs in patients with impaired immunological function [32, 33]. From results of the logistic regression, the more types of Candida spp. detected in dental plaque, the higher likelihood of dentin caries development. Host dental plaque biofilm provided a favorable acidic condition for Candida growth and proliferation, because most yeasts optimally grow at pH 5.5–6 [34]. Hence, patients harboring more Candida species would have a higher risk of demineralization and dissolution of hydroxyapatite.

Nonetheless, the results should be interpreted with caution as one major limitation of this study is the small population size. Only 85 patients were recruited which fell short of the original estimated sample size. Insufficient sample size would lower the statistical power and lead to type II error that might account for the apparent lack of difference in caries increments between the treatment and placebo groups. Furthermore, the length of this study might not be sufficient to fully explore the efficacy of fluoride varnish in caries prevention as caries development is a slow process, despite the fact that SS patients are at high caries risk.

## Conclusions

Results of this randomized clinical trial did not provide clear evidence to support or refute that quarterly applications of fluoride varnish can prevent development of dental caries in people with Sjögren’s syndrome.

## Abbreviations

ADA: American Dental Association
AECG: American-European Consensus Group
CFU: Colony-forming unit
CI: Confidence interval
CO_2_: Carbon dioxide
DMFS: Decayed, missing, filled surface
ICDAS: International Caries Detection and Assessment System
N_2_: Nitrogen
NaF: Sodium fluoride
PBS: Phosphate-buffered saline
pH: Potential of hydrogen
PPDH: Prince Philip Dental Hospital
pSS: primary Sjögren’s syndrome
QEH: Queen Elizabeth Hospital
QMH: Queen Mary Hospital
r_s_: Spearman rank correlation coefficient
SD: Standard deviation
SDA: Sabouraud dextrose agar
SPSS: Statistical package for social sciences
SS: Sjögren’s syndrome
sSS: Secondary Sjögren’s syndrome
SWS: Stimulated whole saliva
UWS: Unstimulated whole saliva
WHO: World Health Organization
