# Erratum to: A randomized, double-blind, placebo-controlled clinical trial of fluoride varnish in preventing dental caries of Sjögren’s syndrome patients

**DOI:** 10.1186/s12903-017-0350-0

**Published:** 2017-03-17

**Authors:** Weini Xin, Katherine Chiu Man Leung, Edward Chin Man Lo, Mo Yin Mok, Moon Ho Leung

**Affiliations:** 1 Dental Care Center, The First Affiliated Hospital of Shantou Medical College, Shantou, China; 2 Prosthodontics, Faculty of Dentistry, The University of Hong Kong, Hong Kong, SAR China; 3 Dental Public Health, Faculty of Dentistry, The University of Hong Kong, Hong Kong, SAR China; 4 Department of Biomedical Sciences, City University of Hong Kong, Hong Kong, SAR China; 5 Department of Medicine, Queen Elizabeth Hospital, Hong Kong, SAR China

## Erratum

After publication of the original article [1], it was noted that one of the author’s affiliation was presented incorrectly. The correct affiliation for Weini Xin should read ‘Dental Care Center, The First Affiliated Hospital of Shantou University Medical College, Shantou, China’. We apologise for any confusion this may have caused.
